# Self-diffusivity, M–S and Fick diffusivity of CO_2_ in Na-clay: The influences of concentration and temperature

**DOI:** 10.1038/s41598-017-05758-3

**Published:** 2017-07-14

**Authors:** Haixiang Hu, Yanfei Xing, Xiaochun Li

**Affiliations:** State Key Laboratory of Geomechanics and Geotechnical Engineering, Institute of Rock and Soil Mechanics, Chinese Academy of Sciences, Wuhan, Hubei 430071 China

## Abstract

Storing CO_2_ in underground saline aquifers is an important way to reduce CO_2_ emission in atmosphere, where gas/fluid diffusion in clay plays a key role in CO_2_ leakage and migration. Various diffusivities, self-diffusivity, Maxwell–Stefan (M–S) diffusivity and Fick diffusivity, in clay interlayer are investigated by molecular dynamics (MD). Self-diffusivity varies with CO_2_ concentration, and reaches the maximum value at 2 molecules/unit-cell. High fluid concentration leads to clay swelling, thereby increasing self-diffusivity. However, the fractional free volume of clay explains the trend of CO_2_ self-diffusivity, which does not decrease with CO_2_ concentration monotonously but reaches the maximum when CO_2_ concentration reaches 2. Displacement distribution of CO_2_ molecules is analysed to explore the microscopic diffusion mechanism, which is characterised by logarithmic normal distribution. The mean value of such distribution further explains the self-diffusivity dependence on CO_2_ concentration. M–S and Fick diffusivities of CO_2_ are calculated by MD for the first time, both of which increase with increasing CO_2_ and H_2_O concentration and temperature. Based on self-diffusivity and M–S diffusivity, a quantity representing the coupling strength between CO_2_ molecules is presented; it increases firstly with CO_2_ concentration but begins to decrease when CO_2_ concentration is beyond 2.

## Introduction

Carbon dioxide (CO_2_) storage in underground saline aquifers is a promising way to reduce CO_2_ emission in the atmosphere. This strategy provides long-term and large-scale storage of CO_2_. Clay minerals^1^, such as illite, chlorite, kaolinite and montmorillonite (MMT) play an important role in this process. On the one hand, owing to their porous (layered) structure, the clay minerals have remarkable capacity of adsorbing CO_2_^2–4^. They provide a large amount of space for storing CO_2_^5^. On the other hand, clay is almost impermeable, and therefore, the clay-enriched cap rocks show excellent sealing ability to retain injected CO_2_^6–8^. Gas leakage and environmental impact are the main problems found after risk assessment of CO_2_ storage. These problems are closely related to fluid (gas) transportation in clay. Gas diffusion in clay is the most important method for gas transportation because of the extremely low permeability of clay.

Many experimental studies have focused on the structure and interaction of CO_2_ and clay mineral^9–12^. Loring *et al*. examined that CO_2_ can migrate the interlayer region of MMT based on the *in situ* X-ray diffraction, magic-angle spinning in nuclear magnetic resonance spectroscopy and attenuated total reflection infrared spectroscopy^10, 13^. Giesting *et al*. investigated the impact of CO_2_ absorption on Ca-exchanged MMT expansion under different CO_2_ pressures^14^. In addition, quasi-elastic neutron scattering experiments on hydrated clays have shown that hydrated cation diffusion mobility is probably a complex dynamic process, and the diffusion coefficients of the exchangeable cations were estimated^15^. Kozaki *et al*. determined the apparent diffusion coefficients of Cs^+^ as functions of the temperature^16^. Sánchez *et al*. discussed that the self-diffusivity of water depends on the temperature and ionic strength of different kinds of clays^17^. However, no experiment on diffusivity of CO_2_ in clay was reported.

With the current high-performance computational resources, molecular simulations have become powerful tools for understanding the molecular-scale structural^18, 19^, thermodynamic^20^, mechanical^21^ and dynamic^22–25^ properties of clay. Grand-canonical Monte Carlo (GCMC) method was used to examine the adsorption of CO_2_ with H_2_O^23^, CH_4_^26–28^ and organic molecules^25, 29^ in clay. Yang *et al*. used molecular dynamics (MD) to study the structure and self-diffusion coefficient (SDC) of CO_2_ in uncharged clay-like slit pores^24^. Cygan *et al*. developed CLAYFF force field^30^ for clay and three-site flexible potential modes of CO_2_^31^. Myshakin *et al*. showed that the intercalation of CO_2_ in clay caused the significant changes in the d-spacing and described that the distribution and diffusion of CO_2_, H_2_O and Na^+^ were affected by the number of layers of clay^32–34^. Botan *et al*. found that CO_2_ affects the diffusion of mobile species in clays^23^.

All the above-mentioned MD simulations mainly investigated the SDCs. Two important diffusion coefficients that are closely related to gas transportation, namely, Maxwell–Stefan diffusion coefficient (MDC) and Fick diffusion coefficient (FDC), have not been investigated. In fact, FDC, instead of SDC, is usually experimentally measurable and is used as parameter input in macroscopic gas transportation formulation, which predicts transportation of underground injected CO_2_. In this work, a series of MD simulations are performed to explore various diffusion coefficients of CO_2_ in clay. The effects of gas concentration, water concentration and temperature on SDC, MDC and FDC are discussed in detail.

## Methods

### Theory of diffusion

As stated above, diffusion coefficients mainly have three types, SDC, FDC and MDC, which are useful in characterising molecular transport and mobility within porous materials. Self-diffusion means random motions or mixing of particles in the thermodynamic equilibrium. In MD simulation, SDC, *D*_*iself*_, of component *i* is computed by Einstein equation^35^:

1 $${D}_{iself}=\frac{1}{2d{n}_{i}}\mathop{\mathrm{lim}}\limits_{t\to \infty }\frac{d}{dt}\sum _{l=1}^{n}{({r}_{l,i}(t)-{r}_{l,i}(0))}^{2},$$

where *n*_*i*_ is the number of the molecules of component *i*, *d* is the dimension of the system and *r*_*l*_,_*i*_(*t*) is the position vector of molecule *l* at time *t*.

FDC is the particle motions driven by chemical or concentration gradient, resulting in the net mass transport. Fick’s law of diffusion defines the transport diffusivity, *D*_*iFick*_, as the proportionality factor between the flux *N* and the concentration gradient:

2 $$N=-{D}_{iFick}\nabla c.$$

Chemical potential gradient is considered as the fundamental driving forces for diffusion^36–40^. For single-component diffusion in porous material, the transport equation can be expressed as follows:

3 $${N}_{i}=-{L}_{i}\nabla {\mu }_{i}.$$

where *μ*_*i*_ is the chemical potential of gas *i*. Moreover, *L* is obtained from the MD simulations using^41^ the following:

4 $${L}_{i}=\frac{1}{2dV{k}_{B}T}\mathop{\mathrm{lim}}\limits_{{\rm{\Delta }}t\to \infty }\frac{1}{{\rm{\Delta }}t}\langle {(\sum _{l=1}^{{n}_{i}}({r}_{l,i}(t+{\rm{\Delta }}t)-{r}_{l,i}(t)))}^{2}\rangle $$

where *V* is the clay volume, *k*_*B*_ is the Boltzmann constant and *T* is the temperature. The content in <…> represents an average on the cross-displacement correlation function (CDCF). The notation < > represents an average on a number of independent runs of same ensembles starting from different initial conditioins.

The Maxwell–Stefan (M–S) theory of diffusion was already available from Maxwell^42^ and Stefan^43^, in which gas diffusion is driven by chemical gradient. Using the M–S theory, the following expression can be derived for diffusion of the single component in a nanoporous material:

5 $${N}_{i}=-\rho {{\rm{\Theta }}}_{i,sat}{D}_{iM-S}\frac{{\theta }_{i}}{{k}_{B}T}\nabla {\mu }_{i}\,,$$

where *ρ* is the clay density expressed as the number of unit cells per cubic meter, Θ_*i*,*sat*_ is the saturation loading of the component *i*, *μ*_*i*_ is the chemical potential expressed in joules per molecule and *N*_*i*_ is the molar flux of the component *i* expressed in molecules per square meter per second. The fractional occupancy *θ*_*i*_ are defined as follows:

6 $${\theta }_{i}=\frac{{{\rm{\Theta }}}_{i}}{{{\rm{\Theta }}}_{i,sat}},$$

where Θ_*i*_ is the loading of the component *i*. Combining equation (3) and equation (5), the MDC, *D*_*iM-S*_, is calculated by the following:

7 $${D}_{iM-S}=\frac{{k}_{B}T}{\rho {{\rm{\Theta }}}_{i}}\,{L}_{i}$$

Under isothermal condition, the molecules are considered to move with average velocity subject to a driving force ∇*μ*_*j*_,*μ* = *μ*_0_ + *k*_*B*_ *T* ln *f*, where *f* is the fugacity and ∇*μ* = *k*_*B*_ *T* ∇*f*/*f*. Thus, equation (2) is equivalent to equation (3).

8 $${N}_{i}=-{D}_{iFick}\nabla {c}_{i}=-\rho D{}_{iFick}\nabla {{\rm{\Theta }}}_{i}=-\rho D{}_{iFick}{\rm{\Theta }}_{i}\frac{\partial \,\mathrm{ln}\,{{\rm{\Theta }}}_{i}}{\partial \,\mathrm{ln}\,{f}_{i}}\frac{\nabla {\mu }_{i}}{{k}_{B}T}$$

The FDC and MDC are related by

9 $${D}_{iFick}={D}_{iM-S}{\rm{\Gamma }},$$

where Γ is the thermodynamic factor

10 $${\rm{\Gamma }}={{\rm{\Theta }}}_{i}\frac{\partial \,\mathrm{ln}\,{f}_{i}}{\partial {{\rm{\Theta }}}_{i}}.$$

### MD simulation details

The sodium-saturated Wyoming-type MMT is used as the clay crystal model with unit formula Na_0.75_[Si_8_](Al_3.25_Mg_0.75_)O_20_(OH)_4_^44^, which comprises 2:1 or tetrahedral−octahedral−tetrahedral layers. The substitution of octahedral Al^3+^ by Mg^2+^ and tetrahedral Si^4+^ by Al^3+^ leads to net negative layer charge. The interlayer Na^+^ cations are balanced by these negative changes. Three-dimensional periodic boundary condition is applied to a simulation box comprising 32 (8 × 4 × 1) united cells. Clay model with different number of CO_2_ and H_2_O are allowed to freely swell to their equilibrium d-spacing. All simulated systems are listed in Table 1. Cn (n = 1, 2,…) denotes the system in which the number of H_2_O is fixed, and the number of CO_2_ is represented by n. Hn (n = 1, 3,…) represent systems with different numbers of H_2_O. Tn (n = 1, 3,…) represents systems with varying temperature.

**Table 1:** Simulation parameters of various simulation systems.

Number of CO_2_	Number of H_2_O	Temperature	d-spacing	Short-name
10 (0.331)	160 (5)	313.15	12.50	C1
16 (0.5)	160 (5)	313.15	12.70	C2
32 (1)	160 (5)	313.15	13.50	C3
48 (1.5)	160 (5)	313.15	14.00	C4
64 (2)	160 (5)	313.15	14.50	C5
80 (2.5)	160 (5)	313.15	14.75	C6
64 (2)	32 (1)	313.15	11.90	H1
64 (2)	96 (3)	313.15	13.00	H3
64 (2)	160 (5)	313.15	14.50	H5
64 (2)	224 (7)	313.15	15.50	H7
64 (2)	288 (9)	313.15	17.25	H9
32 (1)	128 (4)	273.15	12.50	T1
32 (1)	128 (4)	313.15	12.50	T2
32 (1)	128 (4)	353.15	12.50	T3
32 (1)	128 (4)	393.15	12.50	T4
32 (1)	128 (4)	433.15	12.50	T5

Numbers in bracket indicate the corresponding CO_2_ or H_2_O concentration in molecules/cell.

In this article, all simulations are performed with the Accelrys Materials Studio software.The CLAFF force field^30, 31^(Cygan, Liang *et al*. 2004, Cygan, Romanov *et al*. 2012) is used^30–32^. The Ewald summation method is applied for the electrostatic interactions. The atom-based summation method is used in the van der Waals interactions with a cut-off of 12.5 Å. Gas diffusions are simulated as follows: Gases are firstly inserted into the above equilibrium configuration by GCMC method to get a new clay-gas cell. The cell is minimised and equilibrated by NPT and NVT. Then, the cell is run in a NVT ensemble (0.5 ns) followed by a long-time (5 ns) NVE run. The trajectories in the NVE run are saved for diffusion coefficient computation. Integration time step is set to 1 fs. Since diffusion coefficients are based on statistics of numerous molecule trajectories, all the diffusivities in this article are computed by averaging on more than 10 indenpent MD runs to get reliable results.Gas concentration, water component and temperature are used to investigate their impacts on gas diffusions.

## Results and Discussion

**Figure 1:**
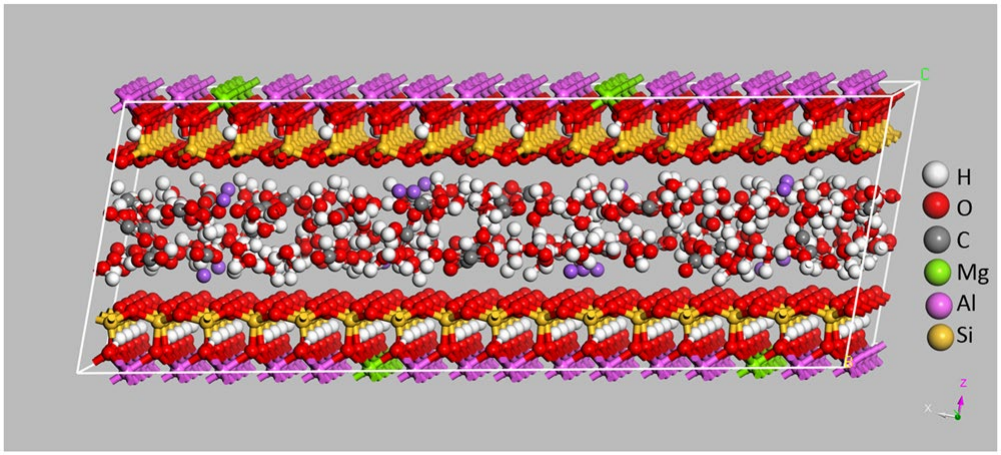
Snapshot of MD simulation of 64 CO_2_ and 160 H_2_O molecules in MMT cell.

MD simulation can be used to obtain not only gas diffusion coefficients but also the microscopic details of the diffusion process, which is helpful to understand underlying diffusion mechanism in a confined space. Figure 1 depicts a simulation snapshot of the diffusion of CO_2_ and H_2_O in MMT clay, from which it is seen that fluid molecules are uniformly distributed in the interlayer of MMT. The direction perpendicular to the walls of interlay is defined as z-axis and the mutually orthogonal x- and y-axes form a plane parallel to the walls. The short length along the z-axis greatly hinders the diffusion of gases along this direction. Thus, only the diffusion behaviour of fluids along the x–y plane is investigated in this study. Unless stated otherwise, the diffusion coefficients in this work refer to the diffusion in the x–y plane.

### Self-diffusion coefficients and its Variation with CO_2_ concentration

Diffusion coefficients are calculated based on the statistical average of the motion of large amounts of particles, and their accuracy relies on the sample size. For the sake of accuracy, each system is run 10 times independently. The resulting MSDs are averaged to calculate SDC. Diffusion coefficients are related to many factors including type and concentration of the gas, size of the pores, temperature and so on. Injection pressure of CO_2_, moisture content and underground temperature are key factors in the geological storage of CO_2_. Therefore, this study focuses on the impacts of CO_2_ concentration, water concentration and temperature on CO_2_ diffusion coefficient.

**Figure 2:**
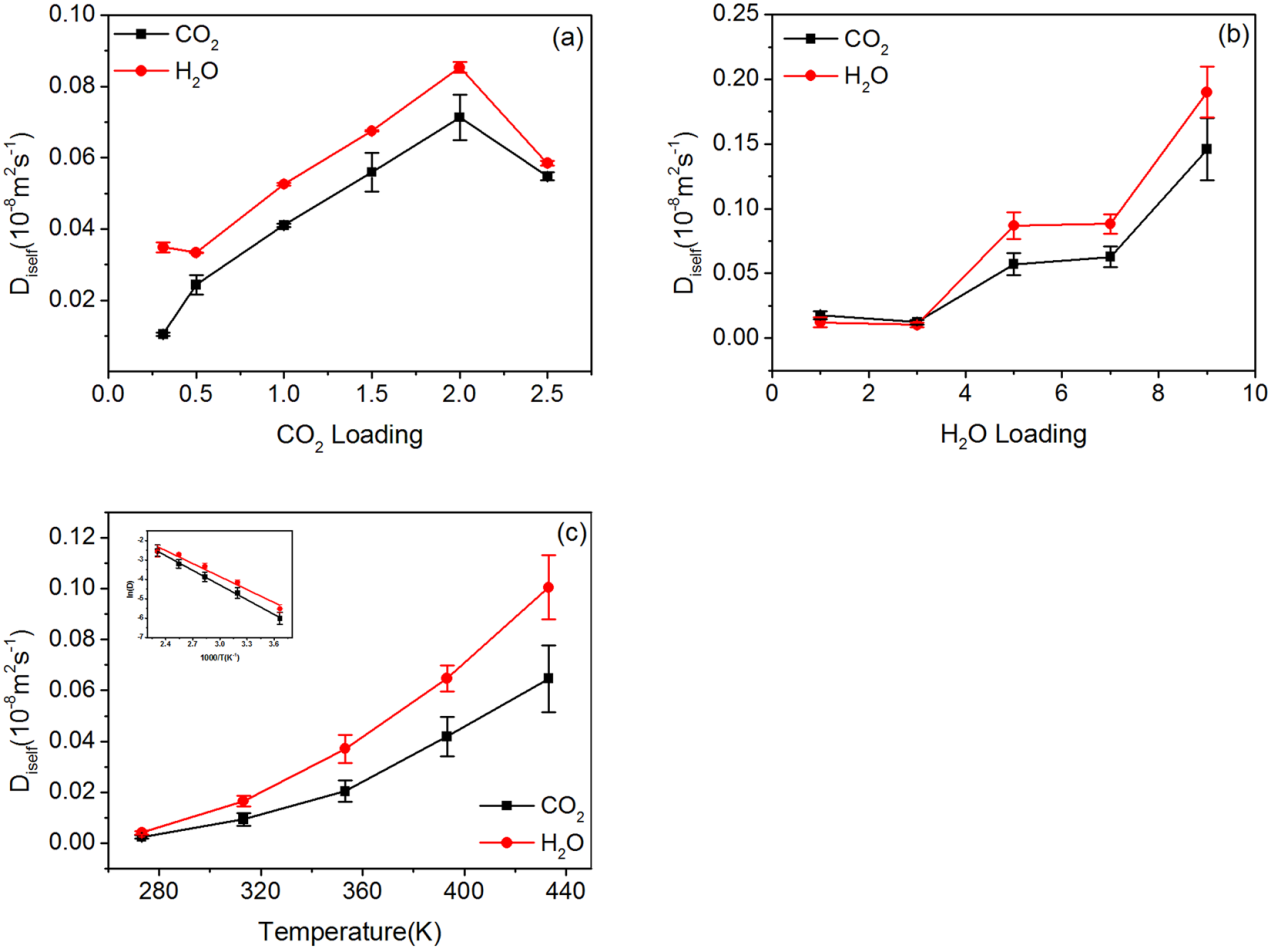
*D*_*iself*_ of CO_2_ and H_2_O in MMT with different systems: (**a**) CO_2_ loading; (**b**) H_2_O loading; (**c**) temperature. Inset of (**c**) shows Arrhenius plot of SDC versus temperature.

Firstly, the influence of CO_2_ concentration on its SDC is studied. The number of water molecules is set to 5 molecules per unit cell and the temperature to 313.15 K (approximately the temperature of 1000 meters underground). The Monte Carlo method is used to insert varying amounts of CO_2_ molecules into the system. The system is then relaxed to equilibrium through NPT and NVT run, with resulting interlay distances (d-spacing) shown in Table 1. The equilibrated system is then subjected to dynamic run in NVE ensemble to compute SDC, as shown in Fig. 2a. With the increase of CO_2_ concentration, the SDCs of CO_2_ and H_2_O firstly increase and then decrease. At the CO_2_ loading value of 64 (C6 system), the SDCs of both reach maximum values. The SDC of H_2_O remains higher than that of CO_2_, which originates from the smaller thermodynamic diameter of H_2_O (2.65 Å) compared with that of CO_2_ (3.3 Å).

**Figure 3:**
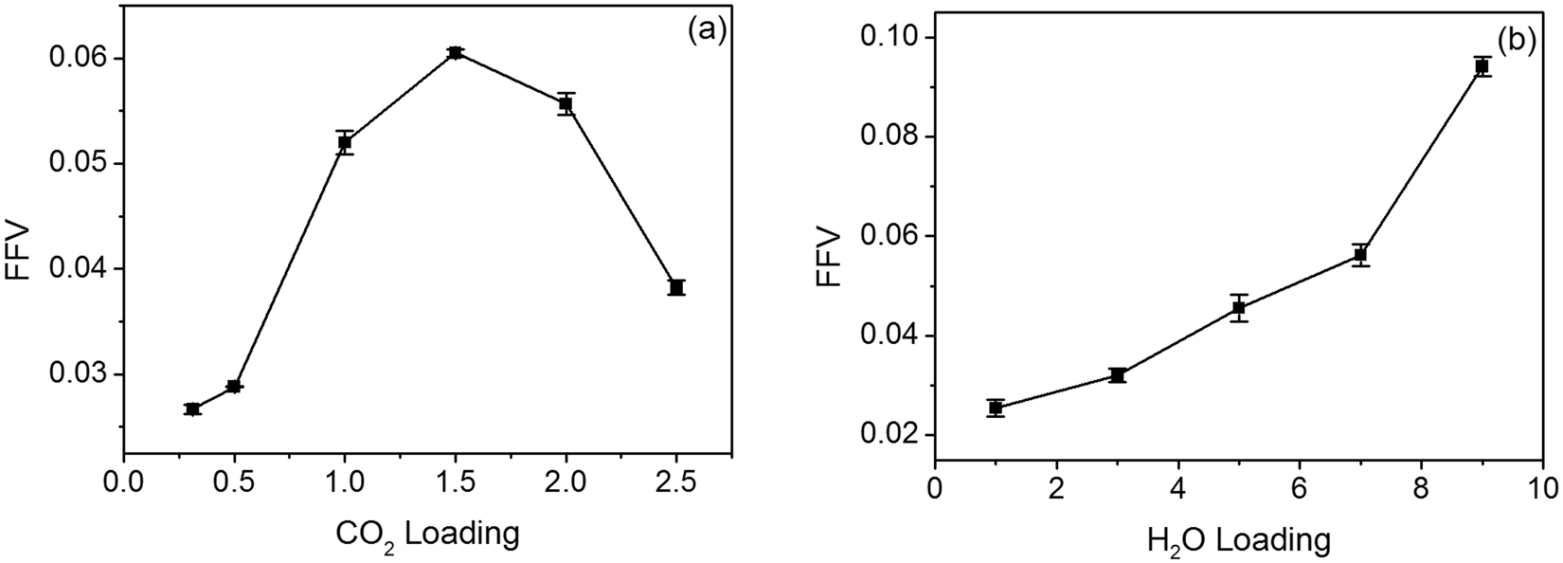
Fractional free volume (FFV) dependence on: (**a**) CO_2_ loading; (**b**) H_2_O loading.

However, many studies had shown that the SDCs of gases in porous media decrease with the increase of their concentrations^36, 38, 45^, which is different from the aforementioned result in Fig. 2a. To explore the cause of the variation of SDC, we further analyse the effect of CO_2_ on the clay structure. As shown in Table 1, the d-spacing of MMT continues to increase with CO_2_ concentration, which could provide fluids with larger space for diffusion, resulting in larger diffusion coefficient. SDC is expected to increase with CO_2_ concentration monotonously. However, our simulations reveal a higher SDC in high-concentration system C6 compared to the low-concentration system C5. To further explore the cause of this unusual SDC variation, fractional free volume (FFV) of the system is defined as the ratio of interlayer space unoccupied by fluids to the total volume of MMT:

11 $${\rm{FFV}}=\frac{{V}_{free}}{{V}_{total}},$$

where *V*_*free*_ is the free volume and *V*_*total*_ is the total volume of the MMT. The FFV can be directly determined by molecular probes^46, 47^. As shown in Fig. 3a, the FFV increases with the CO_2_ concentration firstly and peaks at 64 CO_2_ molecules, which is in accordance with the position of maximum of CO_2_ SDC. This finding indicates two counter-interactions, as follows. Firstly, the increase of CO_2_ concentration could cause the expansion of d-spacing in the system and provide fluids with more space for diffusion. Secondly, increasing CO_2_ molecules occupy more space, thereby resulting in the decrease of free volumes. The fluid molecules become more crowded and diffuse more slowly. Therefore, fluid SDC depends on the free space in the interlayer. At the initial stage of CO_2_ injection, the rapid expansion of d-spacing increases the free volume in the interlayer, which is beneficial for fluid diffusion and increases CO_2_ SDCs. The free volume reaches a maximum at 64 CO_2_ molecules. The free volume decreases despite the continued expansion of d-spacings, which hinders gas diffusion and decreases the SDCs of CO_2_ and H_2_O (Fig. 3a).

### Variation of SDCs with moisture and temperature

Water content varies with the depth of storage aquifer. A MMT system with 2 CO_2_ molecules per unit cell is used to investigate quantitatively the impact of water concentration on the diffusion. Moreover, the temperature is fixed at 313 K. Figure 2b shows the variation of SDCs with H_2_O concentration, where the SDCs of CO_2_ and H_2_O increase with H_2_O concentration. Similarly, the FFVs under different moisture contents can be calculated, as shown in Fig. 3b. The FFV increases with moisture content, showing the same trend as the SDCs. The increase of water molecules increases the free volume in the interlayer and supports the diffusion of fluids.

Temperature is an important factor in the diffusion of gases. As shown in Fig. 2c, increasing temperature can accelerate the thermal motion of molecules and increase their kinetic energy, thereby gradually increasing fluid SDC. Calculations show that the relationship between SDC and temperature follows the classical Arrhenius formula:

12 $$D={D}_{0}{e}^{-\frac{{E}_{a}}{RT}},$$

where *D*_*0*_ is a constant, and E_a_ is the activation energy of diffusion. According to the equation, the activation energy for CO_2_ diffusion in MMT is 20.26 kJ/mol (see inset of Fig. 2c), which is greater than its activation energy of diffusion in pure water 17.8904 kJ/mol^48^. The MMT layers impose restriction on CO_2_ diffusion, causing high activation energy in MMT.

**Figure 4:**
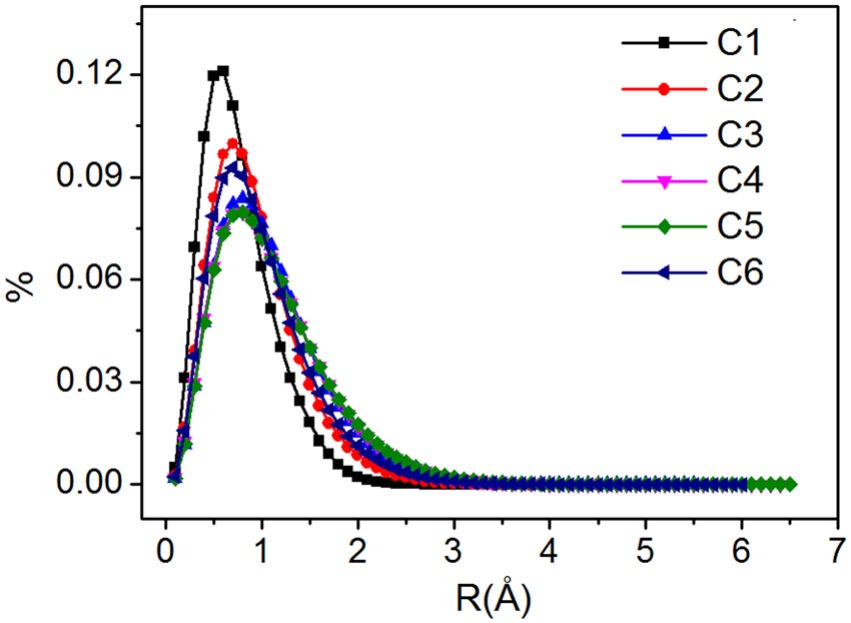
Distributions of displacement, with different systems: (**a**) CO_2_ loading; (**b**) H_2_O loading; (**c**) temperature.

Details such as the paths and displacements of molecules provided by MD simulation can be used to study the microscopic mechanism of gas diffusion. Previous work indicated that the diffusion of gases in porous media (such as polymeric compounds and metal organic frameworks) involves two processes: limited movements within one micropore (small displacements) and jumps among micropores (large displacements)^37^. Layered MMT provides anisotropic confined space without interconnected micropores. Diffusion of gas in such structure is different from random movements of gas in unlimited environment. We further analyse the displacement distribution of CO_2_ molecules, as shown in Fig. 4. All the distributions are seen to have a single peak, and no peak is shown at large displacements. The displacements generally fall within 0.6–1.0 Å. This finding indicates that no inter-pore jumps occur during gas diffusion. Figure 4 also demonstrates that, as CO_2_ concentration increases, the displacement peak (i.e. the prevailing displacement) moves towards the left and becomes wider, reaching the maximum width in the C5 system. This finding implies increases of both the prevailing displacement and the number of fast-speed molecules. Therefore, with the increase of CO_2_ concentration, SDC of CO_2_ increases and reaches a maximum at C5 system. Similarly, the increase of moisture and temperature increases prevailing displacement and peak width, thereby increasing the SDC (as shown in the Figure above). The displacement distributions can be fitted to logarithmic normal distribution function (LNDF):

13 $$y=\frac{{e}^{-\frac{{(-a+\mathrm{log}[x])}^{2}}{2{b}^{2}}}}{d\sqrt{2\pi }x}.$$

**Figure 5:**
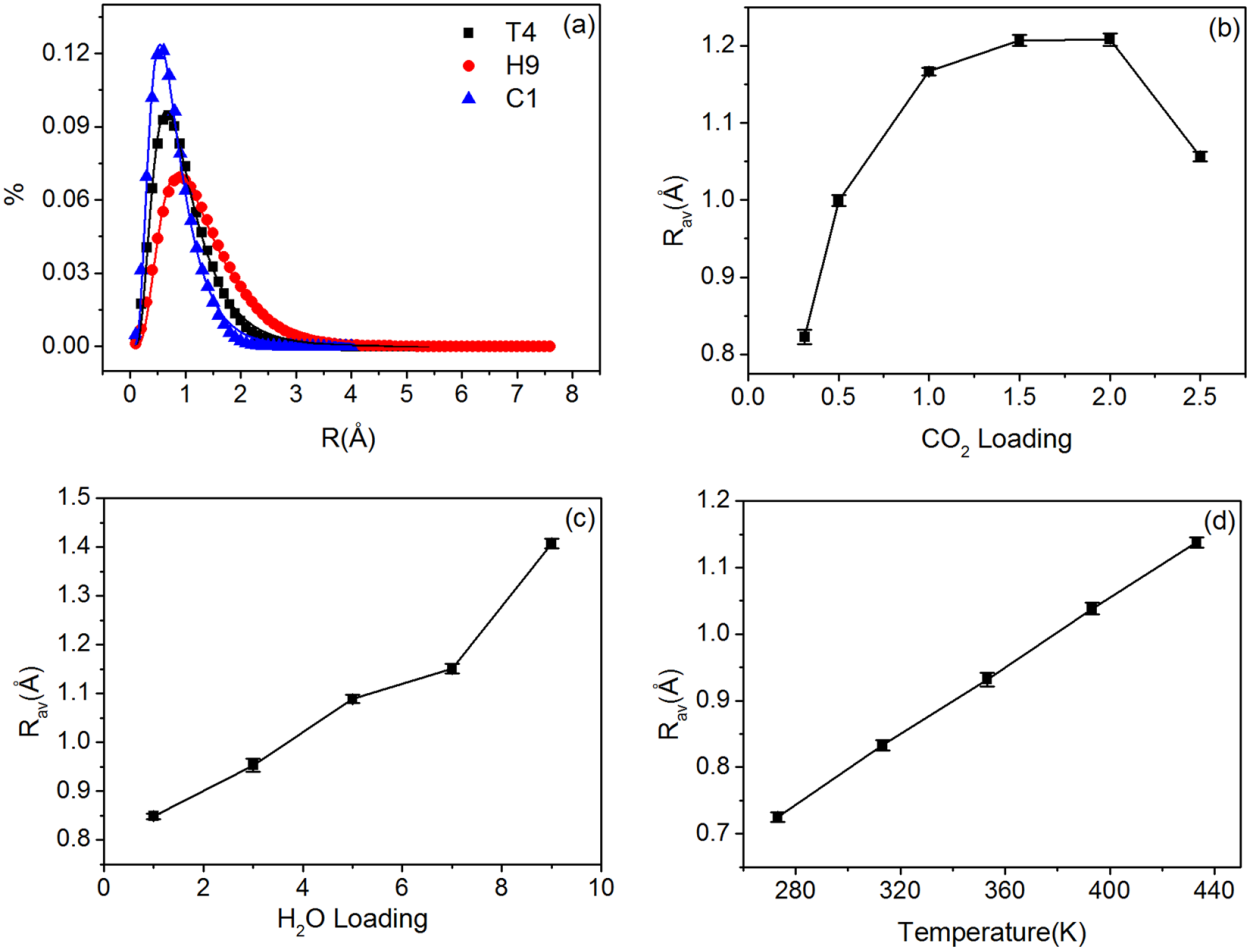
(**a**) Distributions of displacement and the fitted line in logarithmic normal distribution. The average displacement with different systems: (**b**) CO_2_ loading; (**c**) H_2_O loading; (**d**) temperature.

Figure 5a shows some examples, in which curves are observed to be well-fitted to the data (R^2^ > 0.98). To quantitatively examine the relationship between the displacement distribution and SDC, the mean value of LNDF:*R*_*av*_ = ${e}^{(a+{b}^{2}/2)}$, an important characteristic parameter for log-normal distributions, is calculated. The results are shown in Fig. 5b–d. Figure 5b shows the relationship between mean displacement and CO_2_ concentration. In this study, *R*_*av*_ increases with CO_2_ concentration firstly, reaches a maximum at C5 and then decreases, showing the same trend as the SDC of CO_2_. The average displacement of molecules in unit time (1 ps) reflects the relative speed of diffusion, and hence, larger *R*_*av*_ results in faster diffusion speed and a higher SDC. In a word, *R*_*av*_ is positively related to the SDCs of CO_2_ and H_2_O. This finding is further validated by systems with varying moisture contents and temperatures. As shown in Fig. 5c,d, *R*_*av*_ increases with moisture and increases linearly with temperature, causing the increase of SDCs of CO_2_ and H_2_O (Fig. 2).

### Variation of MDC and FDC with concentration, moisture content and temperature

**Figure 6:**
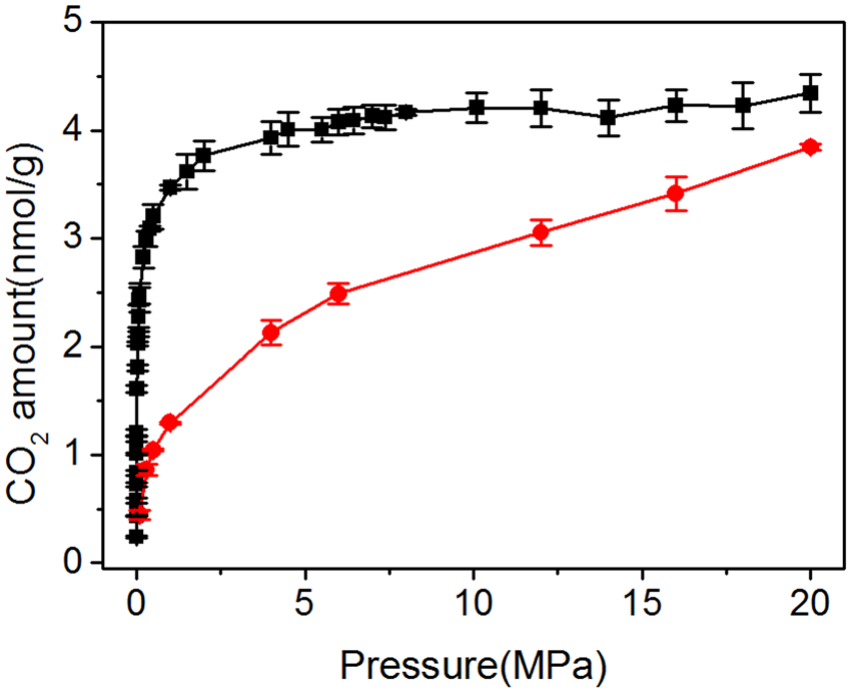
Adsorption isotherms of CO_2_ by conventional GCMC method (black) and flexible adsorption method (red) in C1 system.

To calculate MDC, the Onsager coefficient *L* should be determined using equation (4) in advance. The CDCF can thus be computed from the slope of the curve, which is used to calculate MDC from equation (7) and FDC using equation (9). The thermodynamic factor in equation (9) requires the adsorption isotherm of CO_2_, which can usually be simulated using the GCMC method. The conventional GCMC method assumes a rigid absorbent (μVT ensemble). However, MMT clay swells after adsorbing CO_2_. Swollen MMT can continue to adsorb more gas. Therefore, the conventional GCMC is not suitable for calculating adsorption isotherms of CO_2_ in MMT. In this study, we adopt a new elastic-adsorption approach to simulate CO_2_ isotherm by considering the swelling of MMT. The MMT structure with the initial 224 water molecules is taken as an example (see Fig. 6). Using the conventional GCMC method, we can obtain significantly different adsorption isotherms at different fixed d-spacing values, i.e. the initial and swollen d-spacing. In these isotherms, the curves rapidly approach to saturation at low pressure, and the amount of CO_2_ adsorption is almost kept constant when the pressure rises above 1 MPa (shown by the black line in Fig. 6), which is inconsistent with experimental results^49^. However, the CO_2_ adsorption isotherm obtained by the above elastic-adsorption method increases gradually with the CO_2_ pressure, which is more qualitatively coincident with the Langmuir formula and the above-mentioned experiment results. The maximum adsorption *θ*_sat_ can be obtained by fitting with the Langmuir equation, and then, the thermodynamic factor can be calculated by equation (10). The results show that thermodynamic factor always increases with increasing CO_2_ concentration. For low CO_2_ concentrations, thermodynamic factor is not influenced by H_2_O apparently, whereas at higher CO_2_ concentrations, the thermodynamic factor gradually decreases with H_2_O concentration.

**Figure 7:**
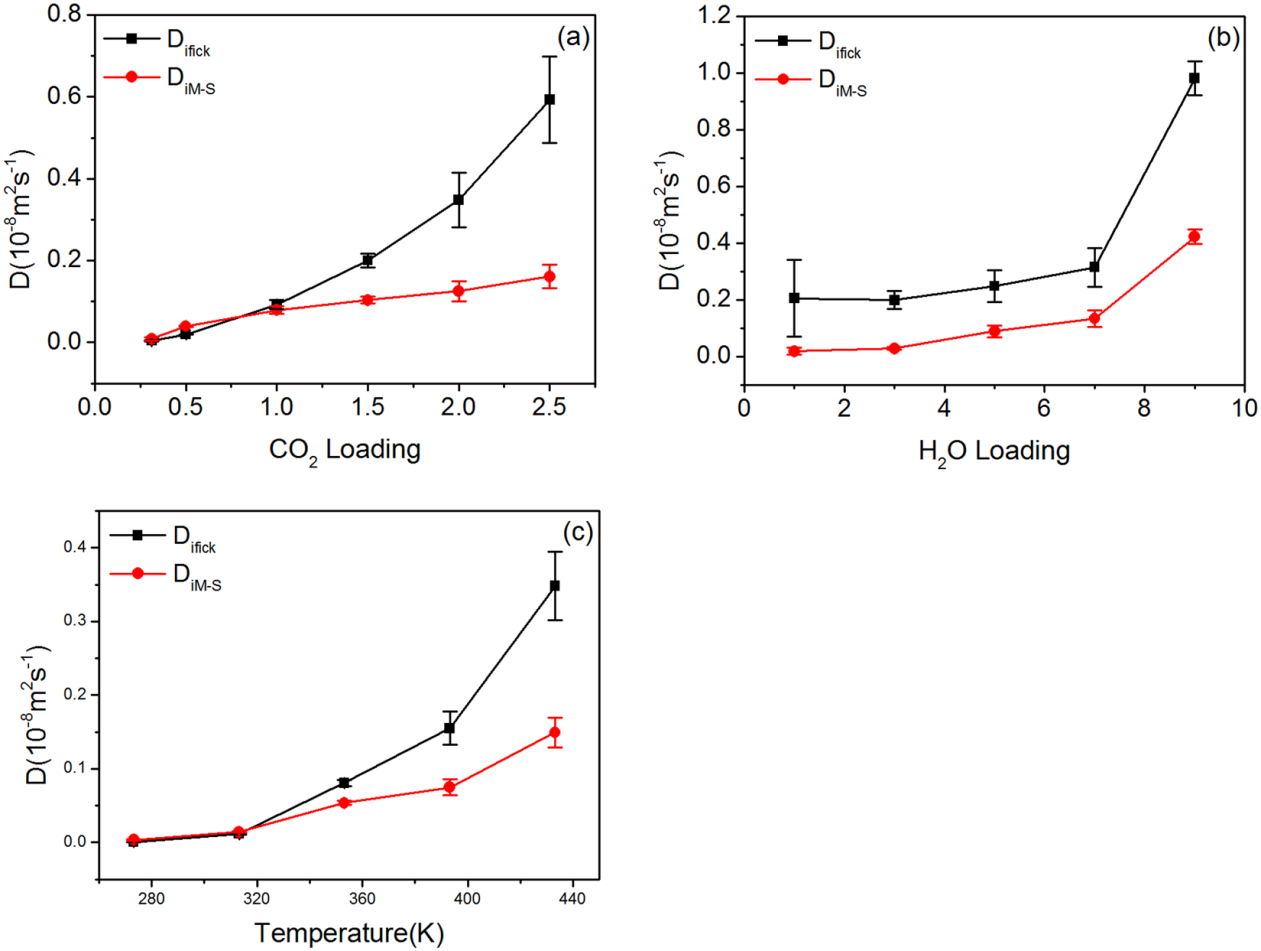
D_iM-S_ and D_ifick_ of CO_2_ in different systems: (**a**) CO_2_ loading; (**b**) H_2_O loading; (**c**) temperature.

The effect of CO_2_ concentration, H_2_O content and temperature on MDC and FDC is also explored. The red dots in Fig. 7 represent the variation of CO_2_ MDC, which increase with CO_2_ concentration, H_2_O content and temperature. The black dots in Fig. 7 represent the FDC. The thermodynamic factor is generally high. Thus, the FDC is usually larger than MDC. Similar to MDC, FDC increases with CO_2_ concentration, H_2_O content and temperature.

The significance differences of CO_2_ diffusivities among CO_2_ concentration moisture content and temperature are calculated as 0.033 (SDC), 0.027 (MDC) and 0.012 (FDC) respectively, indicating the reliablity of the above results.

### Relationship between SDC and MDC: The coupling effect of CO_2_ molecules

Krishna and Paschek *et al*. proved that the difference between MDC and SDC is caused by the self-exchanging coupling between molecules of a diffusing gas^40^. The strength of the coupling between gas molecules can be represented by the self-exchange coefficient *D*_*ii*_, which is calculated by the following formula:

14 $$\frac{1}{{D}_{iself}}=\frac{1}{{D}_{iM-S}}+\frac{{\theta }_{i}}{{D}_{ii}}.$$

**Figure 8:**
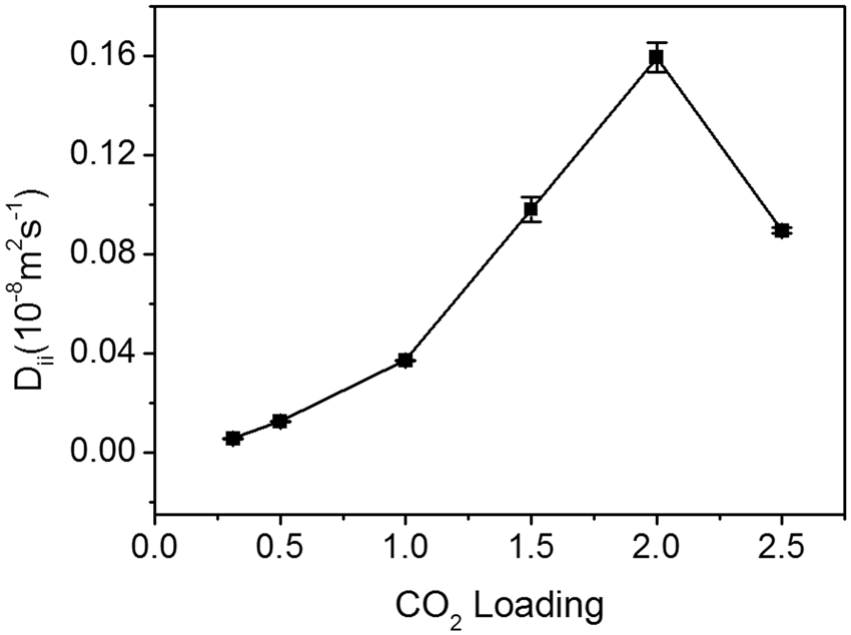
The dependence of *D*_*ii*_ on CO_2_ loading.

A lower value of *D*_*ii*_ indicates stronger correlation and vice versa. The results of Krishna *et al*. indicated that *D*_*ii*_ is significantly influenced by the concentration of the gas: Higher concentration leads to stronger correlation^40^. Using the aforementioned SDC and MDC results, the impact of CO_2_ concentration on *D*_*ii*_ is shown in Fig. 8. With increasing CO_2_, *D*_*ii*_ firstly increased and then decreased, reaching the maximum at 2 molecules/unit cell. *D*_*ii*_ increases with increasing CO_2_ molecules, thereby leading to weak correlation among CO_2_ molecules. When the CO_2_ concentration exceeds 2, *D*_*ii*_ begins to decrease, resulting in stronger correlation. This result is different from the case described in ref. 40, where *D*_*ii*_ monotonically increased with gas concentration in MOF. The coupling between molecules may have been influenced by two factors. On one hand, the correlation is strengthened by increasing the CO_2_ concentration. On the other hand, the rising concentration causes the MMT to expand, thereby increasing the volume of the layer and weakening the correlation. The final strength of the coupling may depend on the FFV within the MMT.

## Conclusions

In this study, MD is used to study various diffusion coefficients of CO_2_ in MMT clay under varying conditions. With increasing CO_2_ concentration, the SDCs of CO_2_ and H_2_O display a peak, respectively. Water content and temperature are positively correlated with the SDC. Many previous studies show that SDCs of gases in porous media usually decrease monotonically with the gas concentration. To further explain the unusual results, the FFV within MMT and the displacement distribution of CO_2_ are analysed. FFV has an important effect on the diffusion of gas molecules in MMT. Increasing CO_2_ concentration causes the expansion of MMT, thereby increasing the internal FFV of MMT and providing gas molecules with more space for diffusion. This finding explains the initial increase of the fluid SDC in MMT. FFV begins to decrease when the CO_2_ concentration rises above 2, which hinders the gas diffusion and thereby decreasing SDC. Similarly, the increase of water content also causes the MMT to expand and create more free space for CO_2_ diffusion, leading to increasing CO_2_ SDC. The fluid SDC in MMT depends on the FFV of MMT. Furthermore, the displacement distribution of CO_2_ follows log-normal distribution, and the mean of the distribution shows the same trend as the SDC, which provides a good explanation of the effects of CO_2_ concentration, moisture and temperature on SDC from another perspective.

Most previous works focused on the self-diffusion coefficients of gases in MMT. FDCs are of practical importance for the geological storage of CO_2_. In this work, the MDC and FDC of CO_2_ in MMT are investigated by MD for the first time, both of which increase with CO_2_ concentration, moisture and temperature. Using the SDC and MDC, we calculate the self-exchange coefficient *D*_*ii*_, which represents the coupling effect among CO_2_ gas molecules. This coefficient firstly increases with CO_2_ concentration, reaching a maximum with concentration 2, and then decreases, showing the same trend as FFV. This implies that, with increasing CO_2_ concentration, the FFV increases, thereby weakening the correlation between CO_2_ molecules until the CO_2_ concentration reaches 2. Then, the FFV decreases and the CO_2_ correlation coupling strength begins to increase.
